# Comprehensive Knowledge of HIV among Women in Rural Mozambique: Development and Validation of the HIV Knowledge 27 Scale

**DOI:** 10.1371/journal.pone.0048676

**Published:** 2012-10-31

**Authors:** Philip J. Ciampa, Shannon L. Skinner, Sérgio R. Patricio, Russell L. Rothman, Sten H. Vermund, Carolyn M. Audet

**Affiliations:** 1 Vanderbilt Institute for Global Health, Vanderbilt University, Nashville, Tennessee, United States of America; 2 Department of Medicine, Universidade Eduardo Mondlane, Maputo, Mozambique; 3 Division of General Internal Medicine and Public Health, Vanderbilt University, Nashville, Tennessee, United States of America; 4 Department of Pediatrics, Vanderbilt University, Nashville, Tennessee, United States of America; 5 Department of Preventive Medicine, Vanderbilt University, Nashville, Tennessee, United States of America; University of Ottawa, Canada

## Abstract

**Background:**

The relationship between HIV knowledge and HIV-related behaviors in settings like Mozambique has been limited by a lack of rigorously validated measures.

**Methods:**

A convenience sample of women seeking prenatal care at two clinics were administered an adapted, orally-administered, 27 item HIV-knowledge scale, the HK-27. Validation analyses were stratified by survey language (Portuguese and Echuabo). Kuder-Richardson (KR-20) coefficients estimated internal reliability. Construct validity was assessed with bivariate associations between HK-27 scores (% correct) and selected participant characteristics. The association between knowledge, self-reported HIV testing, and HIV infection were evaluated with multivariable logistic regression.

**Results:**

Participants (N = 348) had a median age of 24; 188 spoke Portuguese, and 160 spoke Echuabo. Mean HK-27 scores were higher for Portuguese-speaking participants than Echuabo-speaking participants (68% correct vs. 42%, p<0.001). Internal reliability was strong (KR-20>0.8) for scales in both languages. Higher HK-27 scores were significantly (p≤0.05) correlated with more education, more media items in the home, a history of HIV testing, and participant work outside of the home for women of both languages. HK-27 scores were independently associated with completion of HIV testing in multivariable analysis (per 1% correct: aOR:1.02, 95%CI:0.01–0.03, p = 0.01), but not with HIV infection.

**Conclusions:**

HK-27 is a reliable and valid measure of HIV knowledge among Portuguese and Echuabo-speaking Mozambican women. The HK-27 demonstrated significant knowledge deficits among women in the study, and higher scores were associated with higher HIV testing probability. Future studies should evaluate the role of the HK-27 in longitudinal studies and in other populations.

## Introduction

In sub-Saharan Africa, there were an estimated 1.8 million persons newly infected with HIV in 2009, representing an 18% reduction in incidence since 2001 [1]. This progress may reflect, in part, the success of a comprehensive approach to promote HIV prevention that includes increasing knowledge about HIV/AIDS and promotion of healthy behaviors, [1] efforts to prevent mother-to-child-transmission of HIV, [2]–[4] and evidence that treatment of those with HIV infection prevents transmission [5]. In many settings in sub-Saharan Africa, the reduced rate of HIV infection is associated with behavioral changes such as increased condom use, fewer sexual partners and delayed sexual debut among young adults [6]. Improved awareness and knowledge about HIV transmission is often cited as one of the key factors behind these changes; however, it is estimated that only 34% of young adults worldwide have accurate knowledge of HIV, well below the target of 95% set by the United Nations [1], [6]. Ensuring that individuals have the knowledge necessary to protect themselves against HIV infection is an explicit part of the Millennium Development Goal 6: Combat HIV/AIDS, malaria, and other diseases [7].

Accurate knowledge about HIV/AIDS is an important, though insufficient, component of an individual's adoption of healthy behaviors [8]. Understanding the interaction among HIV knowledge, attitudes, and health behaviors for those in high-prevalence settings may improve efforts to promote healthy preventive behaviors, yet research to better understand these relationships in low-income settings such as sub-Saharan Africa has been limited [9]–[20]. Three studies that examined the relationship between HIV knowledge and HIV testing showed mixed results [10]–[12]. A single study of women living in rural Ethiopia found that higher HIV knowledge was associated with more self-efficacy for condom use, more perceived vulnerability to HIV infection, and more intent to use condoms [13]. The lack of an established standard for measurement of HIV knowledge in sub-Saharan Africa has been a factor limiting research to better understand these issues; most published studies used an *ad hoc* questionnaire or interview to ascertain HIV knowledge and do not report the specific items or psychometric properties of the measure, limiting the reproducibility and comparability of findings [10], [12]–[23]. Other studies examined data from Demographic and Health Surveys (DHS) to estimate HIV knowledge in various sub-Saharan populations; [24]–[29] this approach allowed comparison of findings across geographical settings but was limited by the relatively narrow focus of the HIV knowledge-related topics assessed by the DHS, which focused on a few sexual transmission risk-factors, misconceptions about HIV transmission, and mother-to-child transmission risk [24]. One study adapted a scale validated for comprehensive HIV knowledge measurement in the US, the HIV-KQ-45, [30] to assess HIV knowledge of university students in three countries (US, Turkey and South Africa) [31]. The adapted scale was highly reliable and internally consistent; however the instrument did not assess factors that may be more relevant to persons living in sub-Saharan Africa [31].

In Mozambique, the prevalence of HIV infection was 11.5% in 2009 [32]. Despite the nearly universal recognition of HIV/AIDS, only 31.8% of women age 15–49 living in Mozambique have accurate, complete knowledge as measured by five items that assessed basic HIV-related concepts from a DHS survey in 2009 [33]. Few studies have explored factors associated with HIV knowledge or investigated the relationship among HIV knowledge and HIV-related behaviors in Mozambique using *ad hoc* knowledge measures [20], [22], [34]. With the aim of developing a comprehensive measure of HIV knowledge suited for a sub-Sahara African context, items from three existing instruments were adapted, [24], [30], [35] and pooled with original items to comprise the HIV Knowledge 27 Scale (HK-27). The objective of this study was to test the psychometric properties and construct validity of the HK-27 among women completing prenatal care at two clinics in Zambézia Province, and to examine the association between HIV knowledge, past HIV testing, and HIV infection.

## Methods

### Ethics Statement

The study protocol was reviewed and approved by the National Committee of Bioethics for Health in Mozambique (Comité Nacional Bioética Para a Saúde) and the Institutional Review Board of Vanderbilt University. No financial incentive was provided to participants, and written informed consent was obtained for all study participants that included permission to review medical records relating to HIV testing.

### Study Context

Zambézia Province is located in central Mozambique, is predominantly rural, and had an estimated adult HIV prevalence of 12.6% in 2009 [32]. During pregnancy, 58% of women in Zambézia seek prenatal care at least once [36]. HIV counseling, screening, and information regarding mother-to-child transmission are routinely offered to all women seeking prenatal care, and those who are HIV infected are offered antiretroviral prophylaxis or combination antiretroviral therapy (cART) when eligible by national guidelines [37].

### Development of the HK-27

The HK-27 was developed by adapting items from three existing HIV knowledge scales: the Behavioral Surveillance Survey (BSS) from FHI 360, [35] the Demographic Health Survey-AIDS (DHSAIDS), [24] and the 45-item version of the HIV-Knowledge Questionnaire (HIV-KQ-45) [30]. A brief version of the HIV-KQ-45 (the HIV-KQ-18) has been developed, [38] but was not used for this study. Both the BSS (13 items) and DHSAIDS (9 items) assess knowledge of HIV transmission risk factors, are orally administered, and are nested within the framework of a larger questionnaire designed to measure HIV-related stigma and sexual behavior.

Items measuring HIV knowledge from the HIV-KQ-45, BSS and DHSAIDS were pooled and reviewed by experts in HIV care for those living in resource-limited settings, cultural norms and beliefs surrounding HIV in Mozambique, health literacy, and survey design. Items were excluded if they were judged to be redundant in content, less culturally relevant for the Mozambican population, or poorly constructed. The resulting set of items assessed HIV knowledge across three content domains: general disease knowledge, sexual transmission risk factors, and non-sexual transmission risk factors. Four original items were created to add a fourth content domain, assessing knowledge of HIV treatment. A total of 30 items were included after preliminary review; these were translated into Portuguese by a fluent speaker and back-translated into English to verify the accuracy of the translation. The items were also translated by a trained bilingual translator from Portuguese into a local Mozambican language (Echuabo), a principal local language for southern Zambézia Province [39].

The two sets of translated survey items were assessed for clarity and cultural relevance with cognitive interviews of 32 women (19 Portuguese-speakers and 13 Echuabo-speakers) who were awaiting prenatal care at two Zambézia health centers, in Quelimane and Inhassunge. Interviews were conducted in Portuguese or Echuabo, and took place in the language of choice for each participant. A trained interpreter fluent in Portuguese and Echuabo was used to conduct cognitive interviews of Echaubo-speaking participants. These interviews assessed participants' understanding of each item, ensured translations were accurate, and verified all items included in the scale were culturally appropriate. As a result of the cognitive interviews, three items were discarded because of poor clarity, and the translation of 12 items underwent slight revision to better match local language usage, using the suggestions of interview participants. The final scale included 27 items; each item consisted of a statement for which a participant could respond “agree”, “disagree” or “uncertain”. As with the HIV-KQ-45, the response choice of “uncertain” was included to discourage guessing [30].

### Study Population and Measures

A convenience sample of participants was recruited during prenatal care at two health centers. Potential participants were approached after completing a prenatal care consultation. One site was urban (Quelimane) and served patients who commonly spoke Portuguese, a second site (Inhassunge) was rural and served patients who commonly spoke Echuabo; an effort was made to recruit participants proficient in each language at each site. Women were included if they were 1) pregnant, 2) ≥18 years of age, and 3) spoke either Portuguese or Echuabo as their primary language.

After consent was obtained, women were interviewed in a private area of the hospital. All study measures were administered orally in the language of choice of the participant (Echuabo or Portuguese); a trained interpreter fluent in Portuguese and Echuabo was used to facilitate administration of the HK-27 to Echaubo-speaking participants. Sociodemographic characteristics, including age, number of children, education, and occupation, were ascertained by participant self-report. Each participant was administered the HK-27, after which any participant questions about content were addressed.

Each HK-27 item was scored as correct or incorrect, with responses of uncertain counted as incorrect. HIV knowledge was defined as the percentage of HK-27 items answered correctly for each participant. Media item ownership was ascertained by self-reported ownership of television, internet, or cell phone and ranged from 0–3 items. HIV testing was ascertained by self-report to account for tests done at other clinical sites where medical records were inaccessible. HIV test results were obtained on review of the medical record at the study sites only. All data was recorded on paper and entered into a secure electronic database, REDCap™ [40].

### Data Analysis

Sociodemographic data were reported as medians with interquartile ranges (IQR) or as proportions where statistically appropriate for the total sample, and stratified by survey language. Mean HK-27 scores were tabulated along with standard deviations (SD) for the total sample, and separately by language. The proportions of women who obtained an HIV test and were HIV-infected were also tabulated.

To ensure construct validity of the HK-27 in both languages, psychometric testing and validation of the Echuabo and Portuguese language scales were done separately. To establish construct validity of the HK-27, we hypothesized that higher HIV knowledge would be associated with characteristics that may indicate greater exposure to HIV-related information, such as older age, more children (since pregnant women receive HIV counseling as part of prenatal care), receipt of HIV testing, and ownership of more media items. Other factors that may indicate a higher capacity for understanding HIV-related information (more education, work outside of the home, HIV-negative status) were also included in the assessment of construct validity. We hypothesized that Portuguese-speaking participants would have higher scores than participants who spoke Echuabo. Kuder-Richardson 20 coefficients were generated to test internal reliability of the HK-27. Spearman correlations were calculated to assess the association between HK-27 scores and continuous, nonparametric variables; Wilcoxon rank-sum tests compared HK-27 scores across binomial variables, and Kruskal-Wallis tests compared HK-27 scores for categorical variables.

Multivariable logistic regression models examined the relationship between HIV knowledge and self-reported HIV testing. A second set of logistic regression models tested the association between HIV knowledge and HIV-infection for those with medical records available to review. Multivariable models adjusted for study language and site, maternal work, and travel time to the hospital. Covariates in the multivariable model were chosen *a priori* based on the belief that these factors were related to health care access. All analyses were conducted using STATA™ statistical software package (STATAcorp, Release 11, College Station, TX).

## Results

In June and July of 2011, 472 women completing prenatal care visits were approached at two clinics (274 in Quelimane, 198 in Inhassunge); 348 gave consent to participate, yielding a response rate of 74%. Surveys were conducted in Portuguese (188 participants) and Echuabo (160 participants). The HK-27 took between 7–10 minutes for participants to complete. Women had a median age of 24 years (IQR: 20–28), had little formal education (median 5 years, IQR: 2–10), and worked either inside the home or in agriculture (89%). Most (85%) reported they had obtained an HIV test in the past; 6% of women who reported HIV testing had been tested at another site. Of the 275 women who had undergone HIV testing at one of the study sites, 34% had HIV infection. Compared with the Portuguese speaking participants, Echuabo participants reported less education, had fewer participants report work outside the home, had more travel time from home to the clinic, reported more HIV testing, and had lower rates of HIV infection (**Table 1**).

**Table 1:** Characteristics of 348 women seeking prenatal care in Zambézia, Mozambique.

Characteristic		Survey Language	
	Total (N = 348)	Portuguese (N = 188)	Echuabo (N = 160)
**Median age (IQR)**	24 (20–28)	23 (20–28)	24 (20–28)
**Median # of children**	2 (1–3)	1 (1–2)	2 (1–4)
**Median years of education (IQR)**	5 (2–10)	8 (5–10)	3 (0–4)
**Mean travel time (min.) to hospital (SD)**	53 (46)	41 (35)	67 (53)
**Educational attainment, %**			
None	58	32	88
Primary School	22	32	10
Secondary School	17	30	2
University or higher	3	6	0
**Maternal work, %**			
Domestic work	34	54	9
Agriculture	55	27	89
Teacher	6	11	1
Other employment outside home	5	8	1
**Study site, %**			
Quelimane	51	89	6
Innhasunge	49	11	94
**Mean HK-27 score (SD)**	56 (24)	68 (19)	42 (23)
**Accessed HIV testing, %**	85	83	88
**HIV positive, % (N = 275)**	34	39	30

Performance on the HK-27 was higher for Portuguese speakers (mean score: 68%, SD: 19) than for Echuabo speakers (mean score: 42%, SD: 23) (p<0.001). Internal reliability was strong for both scales: the Kuder-Richardson coefficient was 0.8 for the Portuguese language scale and 0.9 for the Echuabo language scale. **Table 2** lists the psychometric performance of each individual HK-27 item by survey language. On the whole, performance was highest on items that tested knowledge of transmission through vaginal intercourse (91% correct for Portuguese speakers, 75% correct for Echuabo speakers) and having multiple sexual partners (81% correct for Portuguese speakers, 72% correct for Echuabo speakers). Three of four women correctly reported that a blood test could identify HIV infection (77% of Portuguese speakers and 71% of Echuabo speakers). Performance on items that assessed knowledge of mother-to-child transmission was mixed; for instance while 85% of Portuguese speaking women correctly answered that vertical transmission could be prevented with medication, only 51% of Echuabo speakers correctly responded. The vast majority of participants in both language groups incorrectly answered an item that tested knowledge of transmission through oral sex (20% correct for Portuguese speakers, 12% correct for Echuabo speakers).

**Table 2:** Psychometric properties of the Portuguese and Echuabo HK-27 scales in Mozambican women seeking prenatal care.

HK-27 Item	Portuguese HK-27		Echuabo HK-27	
	*% Correct*	*Item- total ρ*	*% Correct*	*Item- total ρ*
1. HIV and AIDS are the same thing.^*^	47	0.4	14	0.3
2. A person with HIV can look and feel healthy.^*^	52	0.5	14	0.2
3. A cure for AIDS exists.^*^	63	0.3	27	0.4
4. A blood test can tell if a person has been infected with HIV.^*^	77	0.4	71	0.4
5. A person who feels sick from AIDS can feel better by taking medicines^§^	91	0.4	62	0.6
6. A woman who has HIV can give it to her infant during birth^†^	78	0.4	33	0.5
7. A woman who has HIV can give it to her infant while breastfeeding^†^	79	0.4	27	0.4
8. A pregnant woman who has HIV can prevent her baby from becoming infected by taking medicine^§^	85	0.3	51	0.5
9. A person can get HIV by getting an injection with a needle that was already used on someone else.^‡^	90	0.4	58	0.4
10. A person can get HIV by sharing blades.^†^	94	0.4	66	0.4
11. A person can get HIV from mosquito bites.^‡^	48	0.5	19	0.3
12. A woman can get HIV if she has sex with a man who has HIV.^*^	91	0.3	75	0.6
13. A person can get HIV by sharing forks, spoons or cups with a person who has HIV.^†^	75	0.5	27	0.5
14. A person with HIV can cure the infection by taking medicine. ^§^	30	0.2	22	0.3
15. Eating healthy foods can keep a person from getting HIV.^*^	59	0.5	22	0.4
16. Coughing and sneezing spread HIV.^*^	63	0.4	18	0.3
17. A person can get HIV by shaking hands with someone who has HIV.^*^	88	0.5	45	0.5
18. A person can get HIV by a curse.^†^	70	0.5	30	0.4
19. A person who has HIV can use medicine to prevent becoming sick with AIDS.^§^	47	0.3	60	0.5
20. A person can seek protection from a traditional healer to avoid getting AIDS.^†^	83	0.4	52	0.5
21. A man can get HIV if he has vaginal sex with a woman who has HIV.^*^	90	0.3	72	0.6
22. Bathing or washing one's genitals after sex keeps a person from getting HIV.^*^	60	0.5	37	0.6
23. A person cannot get HIV by having oral sex, mouth-to-penis, with a man who has HIV.^*^	20	0.2	12	0.2
24. Having sex with more than one partner can increase a person's chance of being infected with HIV.^*^	81	0.3	72	0.6
25. A man wearing a latex condom during sex can lower his chance of getting HIV.^*^	70	0.3	67	0.6
26. A person with another STD, such as syphilis, is more likely to get HIV.^*^	63	0.4	57	0.4
27. Cleaning of the vagina with soap before or after sex will keep a woman from getting HIV.^*^	54	0.4	33	0.4

Original Sources: * HIV-KQ-45; ^†^Demographic Health Survey: AIDS; ^‡^Family Health International Behavioral Surveillance Survey; ^§^Original to HK-27.

KR-20: 0.9 Echuabo, 0.8 Portuguese.

Misconceptions about HIV transmission were very common, especially for Echuabo speaking participants, a majority of whom incorrectly responded HIV could be spread by shaking hands, sneezing, sharing utensils or by a curse. Many women of both languages incorrectly reported that cleaning the genitals after intercourse was protective against HIV transmission. Only half of Portuguese speaking women and 14% of Echuabo speaking women agreed that a person with HIV infection could otherwise look and feel healthy.

Higher HK-27 scores were significantly correlated (p<0.05) with more education (ρ = 0.7 for Portuguese speakers, ρ = 0.2 for Echuabo speakers) and more media items in the home (ρ = 0.3 for Portuguese speakers, ρ = 0.2 for Echuabo speakers). Higher HK-27 scores were associated with fewer children at home for Portuguese speakers (ρ = −0.2, p<0.02) but not significantly for Echuabo speakers. Higher HK-27 scores were significantly associated with work outside of the home as compared to domestic work/agriculture for women in both language groups. Women who reported obtaining a test for HIV had higher HK-27 scores than women who had not been tested (Mean HK-27 score: 70% vs. 63%, p = 0.03 for Portuguese speakers, 44% vs. 32% p = 0.03 Echuabo Speakers). Women with HIV infection had lower HK-27 scores than those who had tested negative among Portuguese speakers (Mean HK-27 score: 66% vs. 71% p = 0.04); there were no statistically significant differences in HK-27 scores for Echuabo speaking women with positive vs. negative HIV test results (**Table 3**).

**Table 3:** Relationship between HK-27 scores and selected characteristics for 348 Mozambican women.

Characteristic	Portuguese Speakers			Echuabo Speakers		
	N	Correlation with HK-27 Score (ρ)	P-value*	N	Correlation with HK-27 Score (ρ)	P-value*
**Age**	188	0.1	0.5	160	<0.1	0.8
**Years of Education**	188	0.7	<0.001	159	0.2	0.02
**Number of children**	187	−0.2	0.02	160	<0.1	0.9
**Media Items Owned**	182	0.3	<0.001	159	0.2	0.04

Characteristic	N	Mean HK-27 Score (SD)	P-value	N	Mean HK-27 Score (SD)	P-value
**HIV test obtained**						
Yes	156	70 (18)	0.03†	141	44 (23)	0.03†
No	32	63 (19)		19	32 (21)	
**HIV test positive**						
Yes	50	66 (18)	0.04†	43	43 (25)	0.8†
No	79	71 (18)		103	42 (22)	
**Maternal Work**						
Domestic work	99	65 (20)	<0.001‡	14	63 (12)	0.002‡
Agriculture	53	66 (15)		143	40 (22)	
Business	15	74 (15)		2	63 (10)	
Teacher	21	85 (7)		1	59 (−)	

* Spearman rank correlation.

† Wilcoxon rank-sum test.

‡ Kruskal-Wallis test.

Mean HK-27 scores increased with higher educational attainment for both Portuguese and Echuabo speakers (**Table 4**). Portuguese speaking women had higher mean HK-27 scores than Echuabo speakers among those who reported no formal education (55% vs. 40%, p<0.001) and among those who completed primary school only (70% vs. 55%, p = 0.02); there was no statistically significant difference in scores for Portuguese and Echuabo speaking women who reported secondary school education or more (79% vs. 69%, p = 0.2).

**Table 4:** HK-27 scores and educational attainment of 188 Portuguese and 160 Echuabo-speaking women.

Educational Attainment	Portuguese-speakers		Echuabo-speakers		
	*N*	*Mean HK-27 Score (SD)*	*N*	*Mean HK-27 Score (SD)*	*P-value* *
None	59	55 (16)	141	40 (22)	<0.001
Primary School	61	70 (19)	16	55 (25)	0.02
Secondary School or Higher	67	79 (11)	3	69 (9)	0.1
*P-value* †		<0.001		0.002	

* HK-27 scores of Portuguese-speakers vs. Echuabo-speakers (Wilcoxon rank-sum).

† HK-27 scores by educational attainment (Kruskal-Wallis).

In multivariable logistic regression modeling including data from all participants, HK-27 scores were significantly associated with reported HIV testing after adjusting for survey language, study site, travel time, and maternal work (per 1% correct: aOR:1.02, 95%CI:1.01–1.03, p = 0.01). There was no significant association in fully adjusted models between HK-27 scores and HIV infection for the 275 women with test results available to review in the medical record (per 1% correct: aOR:0.51, 95%CI:0.14–1.78, p = 0.3).

## Discussion

The HK-27 is a valid and reliable measure of HIV knowledge for Portuguese and Echuabo speaking women seeking prenatal care in rural Mozambique, demonstrating strong internal reliability and meeting six of eight criteria selected to establish construct validity for the Portuguese instrument and five of eight criteria for the Echuabo instrument. Women in this study responded correctly to an average of 56% of items on the HK-27. Additionally, we found that HIV knowledge was independently associated with HIV testing, such that women that had higher levels of HIV knowledge had a higher likelihood of reporting that they had been screened for HIV in the past. By including relevant items from the BSS, [37] DHSAIDS, [24] and HIV-KQ-45, [30] standardizing item format, and adding original content, the adapted HK-27 instrument builds on the strengths of the individual existing measures, assesses comprehensive HIV knowledge across multiple content domains, and facilitates future HIV research in settings like Mozambique.

This study adds important detail to what is known about the level of HIV knowledge gleaned from population-based public health surveys such as the DHS in high prevalence settings [24], [25]. Our study shows that while knowledge of basic HIV-related concepts is relatively common, especially related to sexual transmission, there are still significant gaps that may have an impact on HIV prevention and care delivery in rural Mozambique. Some areas where knowledge was lacking may have a negative impact on an individual's ability to behave in ways that prevent HIV infection. For instance, the vast majority of women in our sample did not identify oral sex as a possible route of HIV transmission, and a majority incorrectly specified that washing after sexual contact could protect against transmission. Other common misconceptions, like the belief that HIV can be spread by shaking hands or sharing utensils with an infected person, may contribute to stigma towards those who are infected. Lastly, the belief that HIV could be the result of a curse may cause individuals to avoid conventional HIV care in favor of traditional healers, who may encourage practices that spread HIV infection [39], [41], [42].

In this study, women who spoke Echuabo had lower levels of HIV knowledge than their Portuguese-speaking counterparts. Echuabo-speakers as a group had lower levels of education than Portuguese-speakers, possibly explaining the disparity in HIV knowledge between the groups. However, the observation that Echuabo-speakers had lower HIV knowledge compared to their Portuguese-speaking peers with similar levels of educational attainment would suggest that this is an incomplete explanation. Since Portuguese is the language commonly used in the clinical setting to counsel patients and is often also used in public health educational efforts in Mozambique, [43] women who have limited Portuguese language skills may have an especially difficult time assimilating information meant to educate about HIV. Some health care workers who come from other districts or provinces do not speak Echuabo, for example. In our study, all participants were recruited after completing a prenatal care visit; despite this fact, nearly a quarter of Portuguese-speaking women and three-quarters of Echuabo speakers did not correctly identify vertical transmission as a possible route of HIV infection. This finding raises an important quality-of-care issue–specifically, to consider ways that the content and/or delivery of HIV counseling could be improved, especially for those women that do not speak Portuguese [44], [45]. This and other factors that may explain the stark disparities in knowledge between Portuguese and Echuabo-speaking women in this study should be the focus of future intervention.

Women at both study sites reported a high degree of HIV screening relative to estimates for Zambézia Province from 2009 [32]. Since HIV counseling is customarily provided prior to HIV testing, women in the study may have a higher level of knowledge about HIV than in a population with less testing. Women in this study had a higher rate of HIV infection than in some previous estimates for Zambézia Province [32]. Our findings may accurately indicate the local prevalence of HIV infection among women of childbearing age, as other studies suggest, [46] or may indicate that women with HIV infection were more likely to seek prenatal care, have test results available in the record to review, or were willing to participate in the study. Our use of a convenience sample was a limitation of the study and potentially a source of bias. The level of HIV knowledge and the relationship between knowledge and HIV testing reported in this study may not generalize to the wider population of women in Zambézia Province who do not access prenatal care or who declined to participate in the study. Since women without access to care are less likely to have been tested, and are also likely to have lower HIV knowledge than women receiving prenatal care, it is possible our data underestimate the relationship between knowledge and testing.

Other limitations include the cross-sectional design of the study, which limits the ability to define causality in our observed relationship between HIV knowledge and HIV testing. Our findings may not be generalizable to men or to women over childbearing age. Especially for Echuabo participants, our validation analyses were limited by the low level of participant education and limited variation of work type for women in our study. While we enrolled women at two sites, demographic characteristics of women living in Quelimane and Inhassunge were such that most of the Portuguese-speaking participants were enrolled in Quelimane, and most of the Echuabo-speaking participants lived in Inhassunge. While we adjusted for study site in multivariable models that tested associations between HIV knowledge and testing, it is possible that unmeasured confounders related to rural vs. urban settings influenced our findings. Especially in urban Quelimane, women had opportunities to obtain HIV screening in settings separate from where prenatal care was delivered, and the study was limited by the inability to review medical records at other clinical sites, obligating the use of self-reported HIV testing that introduces the possibility of bias. Since illiteracy is very common among women in Zambézia Province, [47] the HK-27 was designed to be orally administered. As such, this study was not designed to test the validity of the HK-27 as a self-administered instrument to measure HIV knowledge in settings where literacy is more common.

Our study introduces an adapted instrument to measure HIV knowledge that is contextually relevant for use in sub-Saharan Africa, highlights some important gaps in knowledge in two sites in rural Mozambique, and demonstrates a potentially important relationship between knowledge and HIV testing. Our experience adapting the HK-27 into two languages, including a traditional language, suggests that the measure could be adapted into other languages to measure knowledge in other settings in sub-Saharan Africa. While the HK-27 was adapted to use in research efforts, there are other potential applications to the HK-27. First, the measure could be used to assess gaps in knowledge on a community or clinic-level to better tailor HIV-related public health efforts to the informational needs of a given population. Second, the measure could be used as an evaluation tool for HIV-related trainings of counselors, community activists, and health staff. Third, a shortened instrument could be given to individual women prior to their prenatal care counseling, and could be used to focus counseling messages to address gaps in knowledge and avoid message redundancy. Finally, although we found preliminary evidence to support the relationship between HIV knowledge and testing, prospective studies are needed to understand exactly how HIV knowledge impacts health behaviors in sub-Saharan Africa.
